# Validation of the Clinical Treatment Score Post–Five Years in Breast Cancer Patients for Predicting Late Distant Recurrence: A Single-Center Investigation in Korea

**DOI:** 10.3389/fonc.2021.691277

**Published:** 2021-06-21

**Authors:** Jun-Hee Lee, Se Kyung Lee, Byung Joo Chae, Jonghan Yu, Jeong Eon Lee, Seok Won Kim, Seok Jin Nam, Jai Min Ryu

**Affiliations:** Division of Breast Surgery, Department of Surgery, Samsung Medical Center, Sungkyunkwan University School of Medicine, Seoul, South Korea

**Keywords:** hormone receptor-positive breast cancer, premenopausal patients, hormone replacement therapy, CTS5, late distant recurrence

## Abstract

**Background:**

Endocrine therapy is administered to hormone-positive breast cancer patients to prevent distant metastasis. It is important to evaluate the risk of recurrence and to determine which patients are viable candidates for such treatment because hormone therapy has side effects that can include postmenopausal symptoms. The Clinical Treatment Score post–five years (CTS5), a simple tool for identifying candidates for endocrine therapy, was recently introduced; however, CTS5 only has been applied in validation studies with postmenopausal women. We aimed to validate CTS5 among premenopausal breast cancer patients.

**Methods:**

We identified patients treated between 1994 and 2014 at Samsung Medical Center in Seoul, Korea, and followed their treatment outcomes for more than 60 months after surgery using clinicopathologic parameters. According to menopausal status, we divided the study population into two groups: pre- and postmenopausal women. After calculating CTS5 values based on some parameters, we stratified the rate of late distant recurrence (DR) and analyzed the correlation between CTS5 value and late DR by risk.

**Results:**

Among 16,904 patients treated surgically for breast cancer, 2,605 with hormone receptor–positive breast cancer who received endocrine therapy were included. Of these, 1,749 (67.14%) patients were premenopausal women, and the median age was 44.00 years. When categorizing study participants according to CTS5-related risk for late DR, 86.79% were categorized as low risk, 5.95% were categorized as intermediate risk, and 7.26% were categorized as high risk. The annual rate of DR was 1.41% for those in the present study and was similar between pre- and postmenopausal participants (1.40 vs. 1.42). Distant metastasis-free survival was not different between the two groups (hazard ratio: 0.817, 95% confidence interval [CI]: 0.547–1.221). The area under the receiver operating characteristic curve at 10 years for premenopausal and postmenopausal patients was 61.75 (95% CI: 52.97–70.53) and 72.71 (95% CIs: 63.30–82.12), respectively.

**Conclusions:**

Although CTS5 was able to predict late DR, it should be applied with caution in premenopausal women. A CTS5 calculator for premenopausal women might be needed to not underestimate the risk of recurrence in Korea.

## Introduction

Endocrine therapy is inevitable for patients with hormonal status–positive breast cancer to prevent local recurrence and distant metastasis (1–3). Generally, patients with estrogen receptor (ER)- or progesterone receptor (PR)-positive breast cancer are treated with adjuvant endocrine therapy for five years after surgical treatment (4, 5). It is important to evaluate the recurrence risk and determine whether to maintain or stop endocrine therapy after five years based on side effects, such as postmenopausal symptoms, and patient quality of life (6–8). Therefore, it is necessary to decide whether to stop or continue endocrine therapy after weighing the side effects of therapy and the risk for recurrence or metastasis of breast cancer.

Recently, *Dowsett* and colleagues introduced a tool called the Clinical Treatment Score post–five years (CTS5) as a scoring system to help decide whether to stop or continue treatment after five years of endocrine therapy using several clinicopathologic parameters including tumor size, nodal status, and histopathologic grade (9, 10). This scoring system was developed using data from the Arimidex, Tamoxifen, Alone or in Combination (ATAC) trial, which included postmenopausal women with ER-positive or ER-unknown early breast cancer (11, 12). The ATAC trial categorized patients into three risk groups (low, intermediate, and high) for estimating the prognostic performance for late distant metastasis.

However, the CTS5 scoring system might not be as effective in Asian countries because there are many more young breast cancer patients than in Western society (13). In prior research, CTS5 was applied to postmenopausal women in the ATAC and BIG 1-98 study cohorts at diagnosis, and the algorithm was not applied to premenopausal patients (11, 14, 15). CTS5 provides a convenient way to predict distant recurrence (DR) but has limitations in extending its use to all ER- or PR-positive breast cancer patients.

In the present study, we aimed to validate the CTS5 score and develop a modified scoring system to predict distant metastasis not only in postmenopausal women, but also in premenopausal women. We used data from a single institution as the validation set and analyzed participants after subdividing them into pre- and postmenopausal groups to differentiate existing CTS5 scores and identify the prognostic value of CTS5 according to menopausal status.

## Patients and Methods

### Study Populations

**Figure 1:**
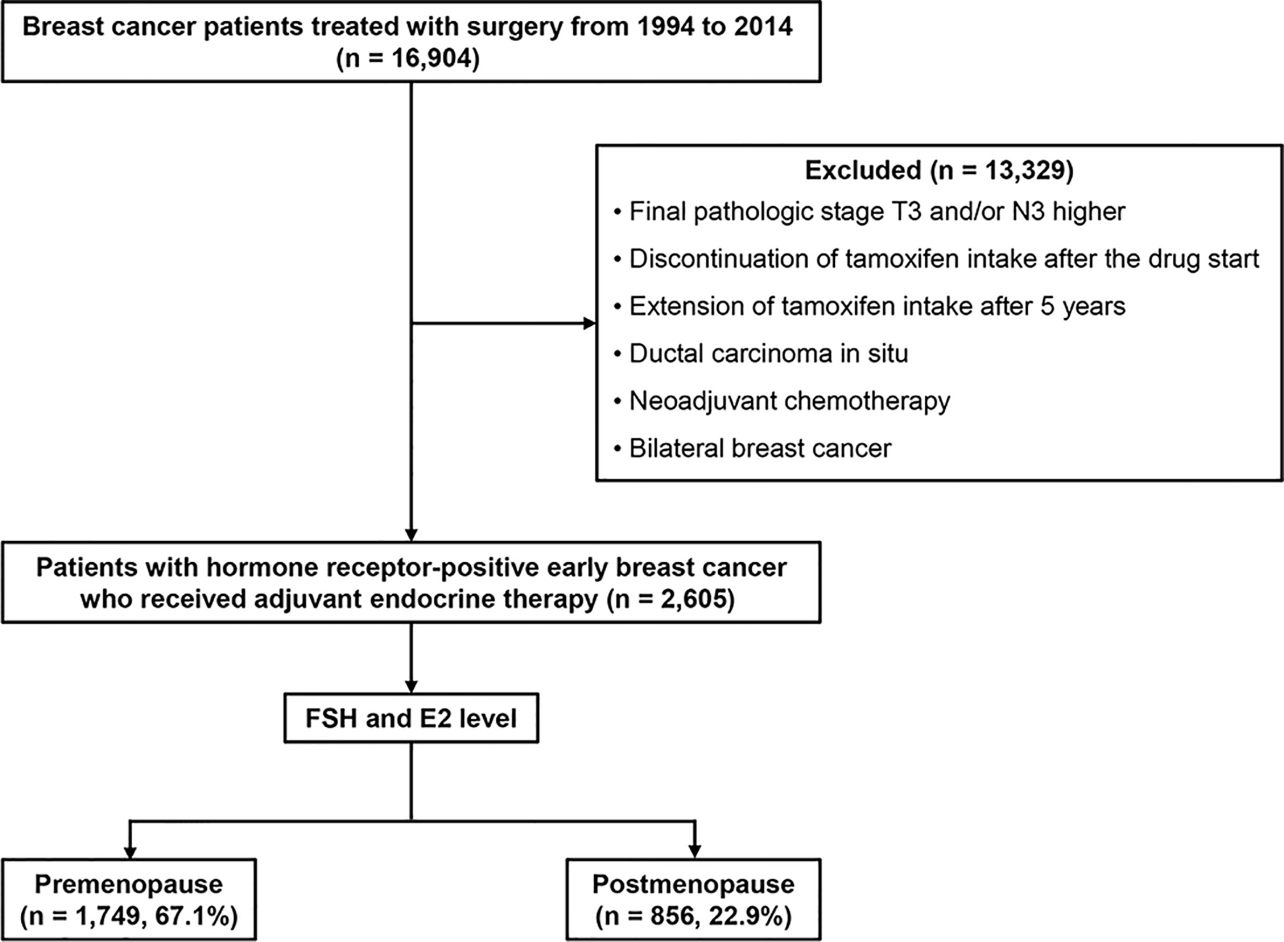
Consort diagram of the study population.

We retrospectively reviewed the medical records of patients who were treated surgically for breast cancer at Samsung Medical Center in Seoul, Korea, between January 1994 and December 2014. Among them, patients with hormone receptor–positive early breast cancer who received adjuvant endocrine therapy and were followed for more than 60 months after surgery were included. We excluded data from women with a final pathologic stage equal to or higher than T3 or N3, ductal carcinoma in situ, or a diagnosis of bilateral breast cancer. Patients who received neoadjuvant chemotherapy also were excluded. Patients with poor drug compliance—those with discontinuation of tamoxifen or aromatase inhibitors such as anastrozole or letrozole intake after starting— also were excluded. Additionally, patients who showed DR prior to five years after diagnosis were excluded from the study cohort. Finally, we excluded patients with extension of adjuvant endocrine therapy after five years (**Figure 1**). According to menstrual cycle period, date of last menstruation, and hormonal test results including follicle-stimulating hormone and estradiol levels, we divided the study population into two groups of pre- and postmenopausal women. Human epidermal growth factor receptor 2 (HER2)-positive patients were included.

### Validation as a Prognostic Tool

Dowsett et al. (9) suggested the use of CTS5 for predicting late DR rates after five years of adjuvant endocrine therapy in patients with hormonal receptor–positive breast cancer. Using the formula CTS5 = 0.438 × nodes + 0.988 × (0.093 × size – 0.001 × size^2^ + 0.375 × grade + 0.017 × age), we validated CTS5 as a prognostic tool for DR onset. We assigned three risk categories in each group of women according to cutoff values of 5% and 10% of DR risk as calculated by CTS5. The cutoff criteria for classifying risk were the same as those of CTS5 values for the combined dataset (ATAC training set and BIG 1-98 validation set).

For survival analysis, the five- to 10-year DR risk was analyzed for each group by Kaplan–Meier plots. The hazard ratio (HR) and 95% confidence interval (CI) of the premenopausal group were calculated and compared to those of the postmenopausal group through univariate analysis. Time-dependent areas under the receiver operating characteristic curve (AUC) at 10 years with 95% CIs were calculated to evaluate matching of the DR rate prediction in between pre- and postmenopausal groups.

### Statistical Analyses

Patient characteristics were compared using the independent t-test for continuous variables and the chi-square or Fisher’s exact test for categorical variables. Univariable and multivariable analyses were conducted using Cox regression analysis models. The five- to 10-year DR risk was estimated using the Kaplan–Meier method. Kaplan–Meier curves, with corresponding log-rank tests, were constructed for DR. Values are reported as mean ± standard deviation (SD) or median with range.

Statistical significance was established at p < 0.05. All statistical analyses were performed using the Statistical Analysis System (SAS) version 9.4 (SAS Institute Inc., Cary, NC, USA) and R Statistical Programming Language Version 2.13.2 (The R Foundation, Vienna, Austria; available at http://www.R-project.org/). The predictability of each CTS5 model for 10-year distant metastasis-free survival was assessed with the time-dependent area under the curve (AUC) and its 95% CI by constructing the receiver operating characteristic (ROC) curve at 10 years post-surgery using R package (16). The present study was approved by the Review Committees (no. 2020-09-143), and work was conducted according to the principles outlined in the Declaration of Helsinki.

## Results

The demographics and baseline characteristics of this study are described in **Table 1**. Among 2,605 patients included in the present study, 1,749 (67.14%) were premenopausal, and 856 (32.86%) were postmenopausal. The median follow-up period was 94.69 months [59.97 – 233.85]. The median age of the premenopausal women was 44.00 years, and that of the postmenopausal women was 56.50 years; overall, the average median age of the study population was 46.00 years. Nodal status, tumor grade, and tumor size were not significantly different between the two groups. A total of 1,902 patients (73.04%) received adjuvant chemotherapy, and more premenopausal women than postmenopausal women received chemotherapy (77.02% *vs.* 64.91%; p < 0.0001). There was no difference between pre- and postmenopausal patients in terms of tumor size, tumor grade, or nodal status. Tamoxifen was administered in 1,481 patients (56.85%) total, 1,325 of whom were premenopausal. Goserelin treatment as a subcutaneous injection of a depot formulation was observed in 159 patients, all of whom were premenopausal. As for aromatase inhibitors, 336 postmenopausal patients (39.25%) received anastrozole, and 278 (32.48%) received letrozole. During the first five years of treatment, 126 patients were switched to aromatase inhibitors, and all 126 patients were classified as premenopausal. Moreover, 110 (4.22%) cases of late DR were recorded, with an annual hazard rate of 1.41% (95% CI: 1.16%–1.70%). There was no significant difference between the two groups in terms of DR cases or annual rate of DR after five years of adjuvant endocrine therapy. The rate for late DR of HER2-positive patients was significantly lower than that of HER2-negative patients (1.64% *vs.* 4.49%; p = 0.0351) (**Supplementary Table 1**).

**Table 1:** Demographic and clinical characteristics between pre- and postmenopausal women.

Characteristic	Total (n = 2,605)	Postmenopausal (n = 856)	Premenopausal (n = 1,749)	p
Age, years				<0.0001
Median	46.00	56.50	44.00	
Interquartile range	42-53	52-62	40-47	
Nodal status				
Negative	1,608 (61.73)	515 (60.16)	1,093 (62.49)	0.1403
1	455 (17.47)	148 (17.29)	307 (17.55)	
2-3	316 (12.13)	112 (13.08)	204 (11.66)	
4-9	226 (8.68)	81 (9.46)	145 (8.29)	
Tumor grade				0.2671
Well	789 (30.29)	242 (29.44)	537 (30.70)	
Moderate	1,279 (49.10)	416 (48.60)	863 (49.34)	
Poor	537 (20.61)	188 (21.96)	349 (19.95)	
Tumor size, mm				0.5903
<10	468 (17.97)	143 (16.71)	325 (18.58)	
10-20	1,229 (47.18)	415 (48.48)	814 (46.54)	
21-30	604 (23.19)	197 (23.01)	407 (23.27)	
31-50	304 (11.67)	101 (11.80)	203 (11.61)	
Chemotherapy				<0.0001
No	702 (26.96)	300 (35.09)	402 (22.98)	
Yes	1,902 (73.04)	555 (64.91)	1,347 (77.02)	
Hormonal therapy				<0.0001
Tamoxifen	1,481 (56.85)	156 (18.22)	1,325 (75.76)	
Toremifene	165 (6.33)	85 (9.93)	80 (4.57)	
Anastrozole	490 (18.81)	336 (39.25)	154 (8.81)^a^	
Letrozole	465 (17.85)	278 (32.48)	187 (10.69)^a^	
Unknown	4 (0.16)	1 (0.12)	3 (0.17)	
GnRH agonist				<0.0001
Goserelin	159 (6.10)	0 (0.00)	159 (9.09)	
Distant recurrence (>5 yrs)				0.8594
No	2,495 (95.78)	819 (95.68)	1,676 (95.83)	
Yes	110 (4.22)	37 (4.32)	73 (4.17)	
Distant recurrence (>5 yrs)				
Annual rate, %	1.41	1.42	1.40	
95% CI	1.16-1.70	1.00-1.96	1.10-1.77	

a 126 patients were switched from tamoxifen to aromatase inhibitors during the first 5 years of treatment.

**Figure 2:**
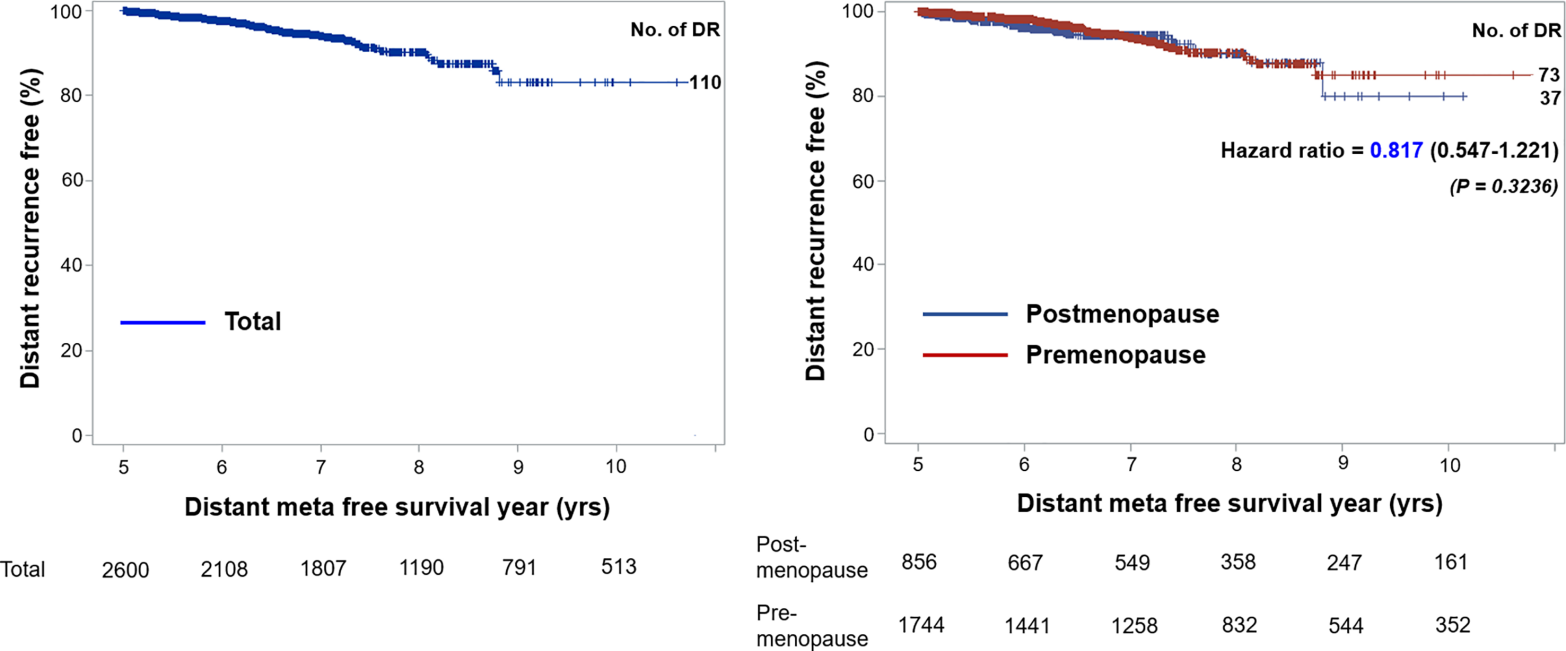
Kaplan-Meier curves of distant recurrence-free survival.

Tumor size, tumor grade, and nodal status were arranged in a separate table according to CTS5 risk category status in pre- and postmenopausal women (**Table 2**). Overall, 86.79% (n = 2,261 patients) were categorized as low risk, 5.95% (n = 155 patients) were categorized as intermediate risk, and 7.26% (n = 189 patients) were categorized as high risk for late DR. Notably, more than 90% of patients in the moderate to poor tumor grade group were categorized high risk, as were all patients with more than two positive nodes. Combined ATAC and BIG 1-98 cohort data and data from participants of this study are compared in **Table 3**. Compared with the ATAC and BIG 1-98 cohorts, there was no difference in tumor size or nodal status. The rate of chemotherapy in our study was more than 70% higher than that of the ATAC and BIG 1-98 cohorts, representing a significant difference, and low risk was identified in more than 80% of patients with the CTS5 score at the time of validation. The HR of five- to 10-year DR risk among premenopausal women was 0.817 (95% CI: 0.547–1.221; p = 0.3236), which was lower than that among postmenopausal women (**Figure 2**).

**Table 2:** Distribution of risk categories according to menopausal status.

Characteristic	Total (n = 2,605)	Low Risk (n = 2,261)	Intermediate Risk (n = 155)	High Risk (n = 189)
**Postmenopausal (n = 856)**				
Tumor size, mm				
<10	143 (16.71)	142 (19.8)	1 (1.61)	0 (0)
10-20	415 (48.48)	375 (52.3)	23 (37.1)	17 (22.08)
21-30	197 (23.01)	142 (19.8)	18 (29.03)	37 (48.05)
31-50	101 (11.8)	58 (8.09)	20 (32.26)	23 (29.87)
Tumor grade				
Well	252 (29.44)	239 (33.33)	8 (12.9)	5 (6.49)
Moderate	416 (48.6)	351 (48.95)	27 (43.55)	38 (49.35)
Poor	188 (21.96)	127 (17.71)	27 (43.55)	34 (44.16)
Nodal status				
Negative	515 (60.16)	515 (71.83)	0 (0)	0 (0)
1	148 (17.29)	135 (18.83)	13 (20.97)	0 (0)
2-3	112 (13.08)	66 (9.21)	33 (53.23)	13 (16.88)
4-9	81 (9.46)	1 (0.14)	16 (25.81)	64 (83.12)
**Premenopausal (n = 1,749)**				
Tumor size, mm				
<10	325 (18.58)	322 (20.85)	1 (1.08)	2 (1.79)
10-20	814 (46.54)	779 (50.45)	16 (17.2)	19 (16.96)
21-30	407 (23.27)	315 (20.4)	44 (47.31)	48 (42.86)
31-50	203 (11.61)	128 (8.29)	32 (34.41)	43 (38.39)
Tumor grade				
Well	537 (30.7)	523 (33.87)	5 (5.38)	9 (8.04)
Moderate	863 (49.34)	751 (48.64)	55 (59.14)	57 (50.89)
Poor	349 (19.95)	270 (17.49)	33 (35.48)	46 (41.07)
Nodal status				
Negative	1,093 (62.49)	1,093 (70.79)	0 (0)	0 (0)
1	307 (17.55)	302 (19.56)	5 (5.38)	0 (0)
2-3	204 (11.66)	140 (9.07)	58 (62.37)	6 (5.36)
4-9	145 (8.29)	9 (0.58)	30 (32.26)	106 (94.64)

**Table 3:** Comparison of combined ATAC and BIG 1-98 cohorts and the present cohort.

Characteristic	ATAC + BIG 1-98 (n = 11,446)	Total (n = 2,605)	Premenopausal (n = 1,749)	p
Nodal status				0.1519
Negative	7,309 (63.86)	1,608 (61.73)	1,093 (62.49)	
1	1,807 (15.79)	455 (17.47)	307 (17.55)	
2-3	1,303 (11.38)	316 (12.13)	204 (11.66)	
4-9	1,027 (8.97)	226 (8.68)	145 (8.29)	
Tumor grade				<0.0001
Well	2,673 (23.35)	789 (30.29)	537 (30.70)	
Moderate	6,215 (54.30)	1,279 (49.10)	863 (49.34)	
Poor	2,558 (22.35)	537 (20.61)	349 (19.95)	
Tumor size, mm				0.6291
<10	2,036 (17.79)	468 (17.97)	325 (18.58)	
10-20	5,562 (48.59)	1,229 (47.18)	814 (46.54)	
21-30	2,599 (22.71)	604 (23.19)	407 (23.27)	
31-50	1,249 (10.91)	304 (11.67)	203 (11.61)	
Chemotherapy				<0.0001
No	8,896 (77.72)	702 (26.96)	402 (22.98)	
Yes	2,550 (22.28)	1,902 (73.04)	1,347 (77.02)	
Distant recurrence (>5yrs)				<0.0001
No	10,746 (93.88)	2,495 (95.78)	1,676 (95.83)	
Yes	700 (6.12)	110 (4.22)	73 (4.17)	
CTS5				<0.0001
Low	4,850 (42.37)	2,261 (86.79)	1,544 (88.28)	
Intermediate	3,620 (31.63)	155 (5.95)	93 (5.32)	
High	2,976 (26.00)	189 (7.26)	112 (6.40)	

**Figure 3:**
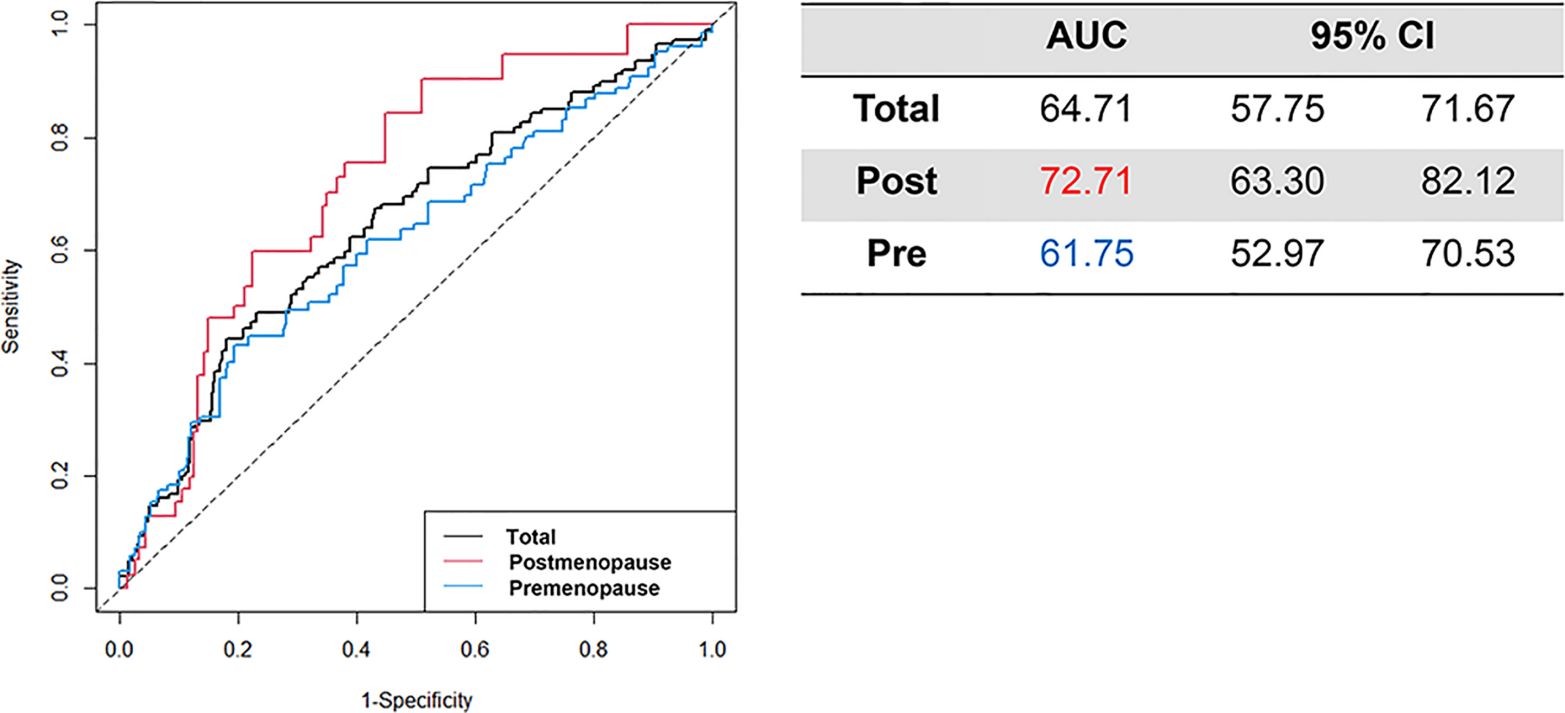
Time-dependent AUC at 10 years post-surgery with 95% CI.

The time-dependent AUC at 10 years is presented with 95% CI value (**Figure 3**). The AUC for all patients was 64.71 (95% CI: 57.75–71.67). Among postmenopausal women, the AUC exceeded the total population AUC at 72.71 (95% CI: 57.75–71.67), whereas that in premenopausal women was 61.75 (95% CI: 52.97–70.53).

**Figure 4:**
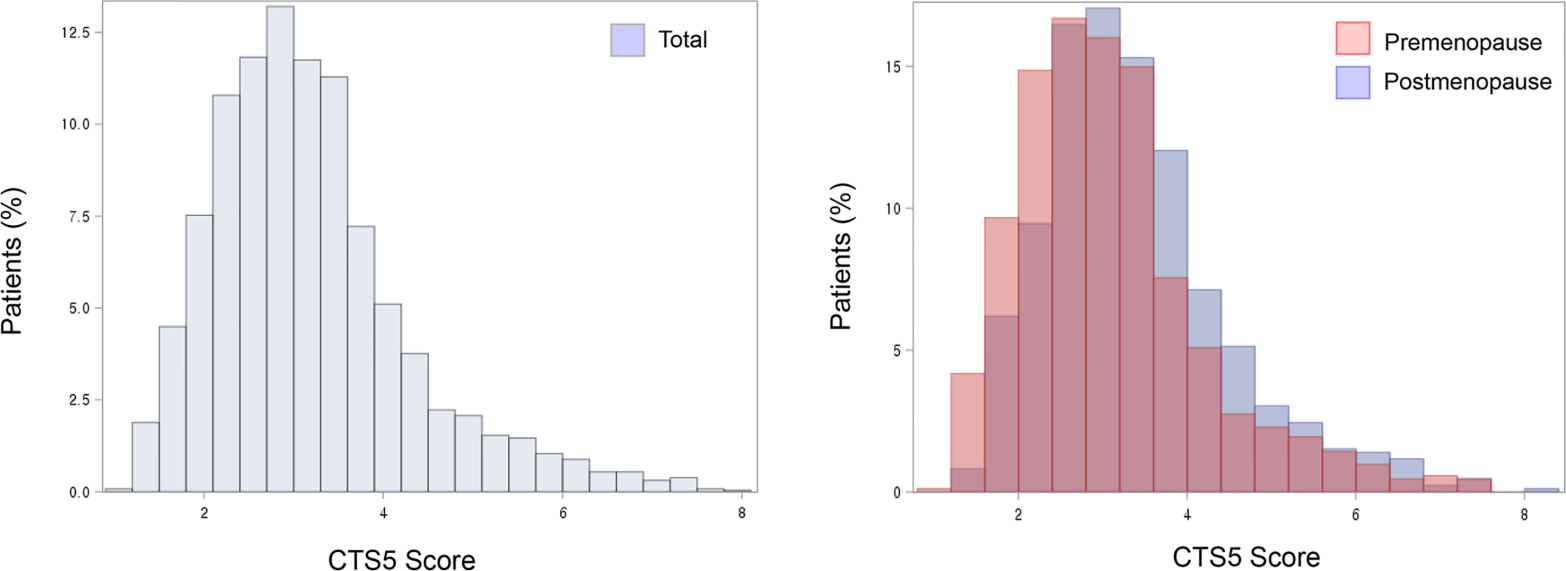
Histogram of CTS5 scores.

Histograms for CTS5 score are shown according to menopausal status with validated prognostic values of CTS5 for risk of DR between five and 10 years (**Figure 4**). Importantly, the premenopausal group included a greater proportion of patients at low risk than did the postmenopausal group. That is, premenopausal women unexpectedly had lower CTS5 scores than postmenopausal women, and this result correlated with the many patients at low risk.

## Discussion

There is a need for prognostic tools that can predict late recurrence rate after five years of endocrine therapy; CTS5 is a useful tool for satisfying this need and supporting clinicians in decision-making regarding extension of endocrine therapy (9, 17). This study is significant in that CTS5 was validated in premenopausal women, who account for the majority of breast cancer patients in Korea. After validation, we found that premenopausal women occupied a large portion of the low-risk recurrence group—in other words, the risk for late DR was underestimated by CTS5 in premenopausal women. Therefore, development of a predictive late DR model for premenopausal women is necessary.

Notably, compared with our study population, the ATAC cohort, used as the training set in CTS5, and the BIG 1-98 cohort, used as the validation set, both included postmenopausal women. Nodal status, tumor grade, and tumor size were similar between the population in our study and the ATAC plus BIG 1-98 cohort. In our study, there was no significant difference in tumor size, tumor grade, or nodal status between pre- and postmenopausal women. There was also no difference in DR or annual DR between the two groups. However, when risk was divided according to the cutoff value of CTS5, patients at low risk were more than twice as numerous relative to the cohorts of the ATAC and BIG 1-98 trials. In addition, the premenopausal group contained a greater number of patients at intermediate and high risk than did the postmenopausal group. Interestingly, the rate of receiving chemotherapy was close to 80% in our study, while about 20% of the ATAC plus BIG 1-98 cohort received adjuvant chemotherapy. For this reason, it is thought that the frequency of adjuvant chemotherapy was high among premenopausal women. The AUC had a greater predictive rate for postmenopausal women than for total patients but was less predictive for premenopausal women than for the entire cohort. In other words, as CTS5 was created based on data of postmenopausal women, there is a high probability that it cannot efficiently be applied to premenopausal women in many Asian countries (18). Premenopausal women are likely to be classified in an underestimated risk group when CTS5 is used, and it is necessary to introduce a new scoring system to address this.

Recently, many studies have analyzed the prognostic value of CTS5. Villasco et al. (19) found that CTS5 has prognostic value in predicting late DR in both pre- and postmenopausal women by testing its clinical validity in a retrospective cohort, while Lee et al. (20) similarly concluded that CTS5 is a good prognostic tool for evaluating the risk of late distant recurrence in both pre- and postmenopausal women using the Ki-67 labeling index and confirmed its prognostic performance in premenopausal women. Although Lee et al. reported that their risk groups presented differences in tumor grade relative to the ATAC and BIG 1-90 cohorts, there was no significant difference in tumor grade between our group and the ATAC plus BIG 1-90 cohort. We think that the reason for the different risk groups might be related to whether or not adjuvant chemotherapy was available.

*HER2* gene amplification is known to have an impact on breast cancer, and the intracellular signaling pathway of estrogen receptors and *HER2* has a complex connection (21). Wang et al. (22) recently suggested that *HER2* status has an effect on late DR in hormone receptor–positive breast cancer. In *HER2*-positive patients, such as those with luminal type B disease, a less effective prognostic value was found in their study. Cases of *HER2*-positive breast cancer are considered high-risk for DR but, due to the development of anti-*HER2*–targeted therapy, it is not effective for the *HER2*-positive model, and a further prediction model is required.

Recently, Noordhoek et al. (23) published a validity and accuracy study of the CTS5 for predicting the rate of late DR in the TEAM and IDEAL trials, arguing the CTS5 overestimates the risk of late DR in high-risk subgroups and suggesting that CTS5 should be used cautiously for interpreting the DR rate among patients at high risk despite its ease of use. Based on these findings, CTS5 must be applied carefully, and unique validation is justified in Korea, where there are many young breast cancer patients.

Various multigene assays have been developed to complement predictions using existing clinicopathologic parameters because of the lack of predictive values for late DR. Among them, GenesWell™ BCT has been validated as a tool to predict late DR (24). In some patients who underwent CTS5 validation, GenesWell™ BCT also was applied, and the CTS5-derived risk group correlated with the gene assay risk group (**Supplementary Figure 1**). However, the use of multigene assays is controversial in predicting late DR because some assays have not been validated, and the importance of clinical risk has been emphasized, especially in multigene panels of patients under 50 years of age (25). In conclusion, balanced application of a clinicopathologic prediction model and a multigene assay should be conducted to predict late DR, and further study is needed for proper selection of patients to receive extended adjuvant endocrine therapy.

The main limitation of this study is that it was a retrospective study conducted in a single center. It is necessary to conduct multicenter studies to further explore the limitations of CTS5, and a modified version of CTS5 should be developed and validated in large sets through multicenter research in South Korea. In addition, it is necessary to accurately stratify risk groups by creating new cutoff values suitable for Korean patients. Accordingly, we plan to create a web-based search tool suitable for use in Korea.

When the CTS5 calculator was developed, HER2-positive patients were included despite the small population. Because the diagnostic technique has not been applied, such as silver-enhanced *in situ* hybridization in our data prior to 2003, it is not clear to describe the HER2 status. Due to development of HER2 gene amplification diagnosis, HER2 status can be accurately described, allowing not only chemotherapy, but targeted therapy to be administered. HER2-positive breast cancer is different from hormone receptor-positive breast cancer in terms of molecular biology, and further study is needed to predict late DR excluding the HER2-positive subtype. In addition, this study did not confirm the difference in CTS5 risk among premenopausal women according to use of GnRH agonists because of the small population (156 patients) of premenopausal patients received GnRH agonists. GnRH agonists have been used to suppress ovarian function in young patients with luminal-type breast cancer and premenopausal patients with high risk for DR due to poor prognosis (26, 27). In the SOFT-TEXT and ASTRRA trials, it was found that the prognosis of young breast cancer patients could be improved depending upon the use of GnRH agonists (28–31). In the future, additional CTS5 evaluation is required according to the use of GnRH agonists in premenopausal women, and it is necessary to evaluate the late prediction rate according to the combination of CTS5 and multigene assay by GnRH agonist use.

CTS5 can be prognostic, but risk evaluation is dependent upon traditional clinicopathologic parameters such as tumor size, tumor grade, and nodal status; it cannot provide customized guidance for each individual. Therefore, its combination with a multigene assay is important for deciding whether to extend endocrine therapy (17, 32). Furthermore, in addition to a clinical calculator, such as CTS5, and a multigene assay, further combination with an immunohistochemistry assay and radiographic imaging (computed tomography, positron-emission tomography, and magnetic resonance imaging) or tumor marker assessments is important for predicting patient prognosis (25, 33).

In conclusion, although CTS5 was created to support decision-making by clinicians about extending adjuvant endocrine therapy in postmenopausal women with positive hormonal receptors, there are limitations in predicting late DR in premenopausal women. For populations of premenopausal women with greater rates of breast cancer, a modified scoring system for late DR prediction is needed so as not to underestimate the recurrence risk.

## Data Availability Statement

The original contributions presented in the study are included in the article/**Supplementary Materials**, further inquiries can be directed to the corresponding author.

## Ethics Statement

The present study was approved by the Review Committees (no. 2020-09-143). The patients/participants provided their written informed consent to participate in this study.

## Author Contributions

J-HL performed literature search, the data analysis, and drafted the manuscript. SL and BC performed revised the manuscript. JY performed literature investigation and reviewing. JEL SK, and SN performed supervision and reviewing. JR designed the concept of article. All authors contributed to the article and approved the submitted version.

## Funding

This study was supported by the Korea Breast Cancer Foundation (no. PHO020413) and the biostatistics team of the Statistics and Data Center, Research Institute for Future Medicine, Samsung Medical Center.

## Conflict of Interest

The authors declare that the research was conducted in the absence of any commercial or financial relationships that could be construed as a potential conflict of interest.
