# Supplementary figures and images for: Validation of the Clinical Treatment Score Post–Five Years in Breast Cancer Patients for Predicting Late Distant Recurrence: A Single-Center Investigation in Korea
Source: Front Oncol. 2021 Jun 21;11:691277. doi: 10.3389/fonc.2021.691277 (PMC8257467; doi:10.3389/fonc.2021.691277)

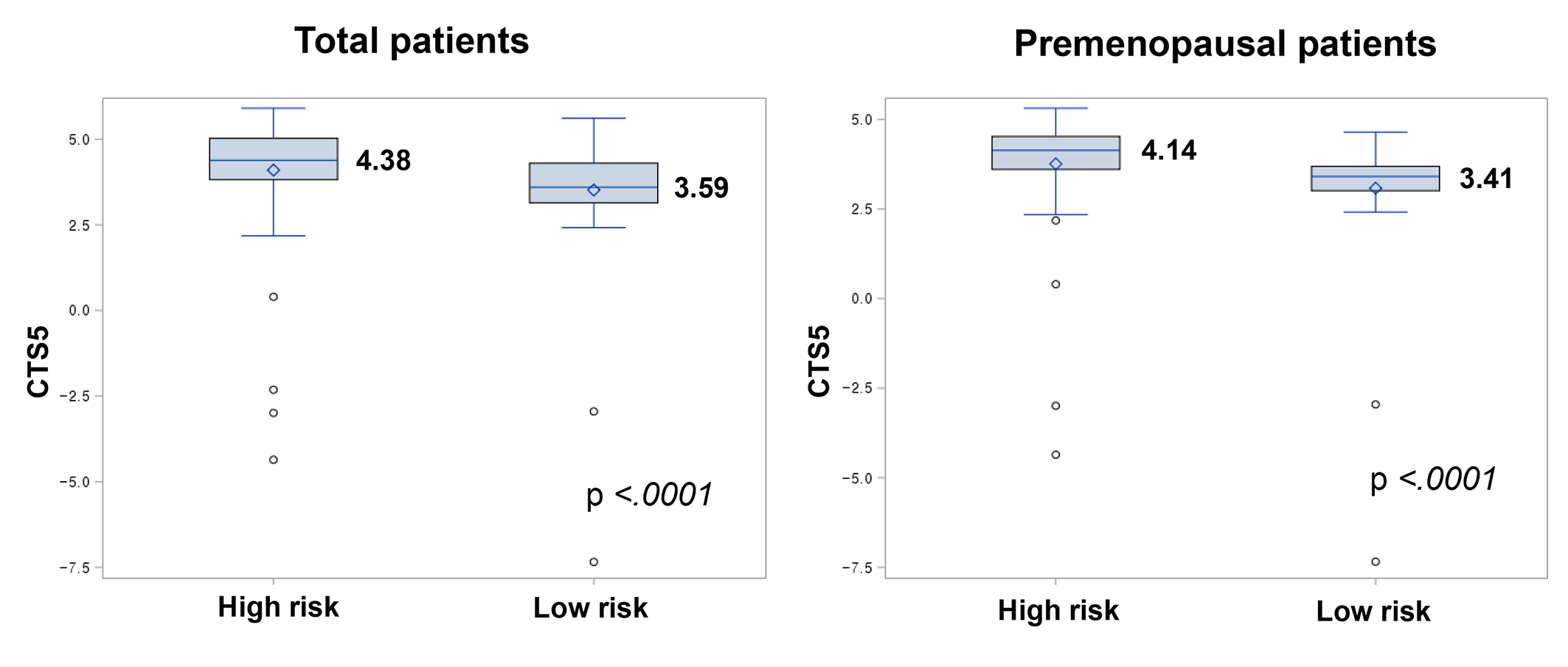

Supplement: Supplementary file 1 [file Image_1.tif]
