# Supplementary material for: Validation of the Clinical Treatment Score Post–Five Years in Breast Cancer Patients for Predicting Late Distant Recurrence: A Single-Center Investigation in Korea
Source: Front Oncol. 2021 Jun 21;11:691277. doi: 10.3389/fonc.2021.691277 (PMC8257467; doi:10.3389/fonc.2021.691277)
Supplement: Supplementary file 2 [file Table_1.docx]

| **Characteristic** | **Total**  **(n = 2,605)** | **HER2 positive**  **(n = 244)** | **HER2 negative**  **(n = 2,361)** | **p** |
| --- | --- | --- | --- | --- |
| Age, years |  |  |  | 0.5801 |
| Median | 46.00 | 46.00 | 46.00 |  |
| Interquartile range | 42-53 | 42-53 | 42-53 |  |
| Nodal status |  |  |  | 0.0223 |
| Negative | 1,608 (61.73) | 143 (58.61) | 1,465 (62.05) |  |
| 1 | 455 (17.47) | 30 (12.30) | 425 (18.00) |  |
| 2-3 | 316 (12.13) | 44 (18.03) | 272 (11.52) |  |
| 4-9 | 226 (8.68) | 27 (11.07) | 199 (8.43) |  |
| Tumor grade |  |  |  | <.0.0001 |
| Well | 789 (30.29) | 18 (7.38) | 771 (32.66) |  |
| Moderate | 1,279 (49.10) | 87 (35.66) | 1,192 (50.49) |  |
| Poorly | 537 (20.61) | 139 (56.97) | 398 (16.86) |  |
| Tumor size, mm |  |  |  | 0.1677 |
| < 10 | 468 (17.97) | 34 (13.93) | 434 (18.38) |  |
| 10-20 | 1,229 (47.18) | 117 (47.95) | 1,112 (47.10) |  |
| 21-30 | 604 (23.19) | 64 (26.23) | 540 (22.87) |  |
| 31-50 | 304 (11.67) | 29 (11.89) | 275 (11.65) |  |
| Chemotherapy |  |  |  | <0.0001 |
| No | 702 (26.96) | 33 (13.52) | 669 (28.35) |  |
| Yes | 1,902 (73.04) | 211 (86.48) | 1,691 (71.65) |  |
| Distant recurrence (>5yrs) |  |  |  | 0.0351 |
| No | 2,495 (95.78) | 40 (98.36) | 2,255 (95.51) |  |
| Yes | 110 (4.22) | 4 (1.64) | 106 (4.49) |  |
| Distant recurrence (>5yrs) |  |  |  |  |
| Annual rate, % | 1.41 | 0.66 | 1.47 |  |
| 95% CI, % | 1.16-1.70 | 0.18-1.68 | 1.21-1.78 |  |

**supplementARY Table 1** | Demographic and clinical characteristics between HER2 positive and negative patients.
